# Semantic content outweighs low-level saliency in determining children’s and adults’ fixation of movies

**DOI:** 10.1016/j.jecp.2017.09.002

**Published:** 2018-02

**Authors:** Andrew T. Rider, Antoine Coutrot, Elizabeth Pellicano, Steven C. Dakin, Isabelle Mareschal

**Affiliations:** a UCL Institute of Ophthalmology, University College London, London WC1E 6BT, UK; b Centre for Mathematics and Physics in Life Sciences and Experimental Biology (CoMPLEX), University College London, London WC1E 6BT, UK; c Centre for Research in Autism and Education (CRAE), Department of Psychology and Human Development, UCL Institute of Education, University College London, London WC1H 0AL, UK; d School of Psychology, University of Western Australia, Crawley, Perth, Western Australia 6009, Australia; e School of Optometry and Vision Science, University of Auckland, Auckland 1010, New Zealand; f Department of Psychology, Queen Mary University, London E1 4NS, UK

**Keywords:** Eye movements, Visual attention, Development, Gaze, Dynamic, Faces, Saliency

## Abstract

To make sense of the visual world, we need to move our eyes to focus regions of interest on the high-resolution fovea. Eye movements, therefore, give us a way to infer mechanisms of visual processing and attention allocation. Here, we examined age-related differences in visual processing by recording eye movements from 37 children (aged 6–14 years) and 10 adults while viewing three 5-min dynamic video clips taken from child-friendly movies. The data were analyzed in two complementary ways: (a) gaze based and (b) content based. First, similarity of scanpaths within and across age groups was examined using three different measures of variance (dispersion, clusters, and distance from center). Second, content-based models of fixation were compared to determine which of these provided the best account of our dynamic data. We found that the variance in eye movements decreased as a function of age, suggesting common attentional orienting. Comparison of the different models revealed that a model that relies on faces generally performed better than the other models tested, even for the youngest age group (<10 years). However, the best predictor of a given participant’s eye movements was the average of all other participants’ eye movements both within the same age group and in different age groups. These findings have implications for understanding how children attend to visual information and highlight similarities in viewing strategies across development.

## Highlights

• Content outweighs saliency when watching films.
• Variance between eye movements decreases with age.
• Gaze patterns show similar (qualitative) strategies across development.

## Introduction

Eye tracking is increasingly being used to try to infer what people are doing (Hayhoe & Ballard, 2005) or thinking (Kardan, Berman, Yourganov, Schmidt, & Henderson, 2015) based solely on how they looked at a visual scene. One advantage of this method over, for example, self-report or psychophysical testing is that it can readily be applied to populations that are difficult to evaluate, from young babies (Jones, Kalwarowsky, Atkinson, Braddick, & Nardini, 2014) to clinical populations, including autistic people (e.g., see Papagiannopoulou, Chitty, Hermens, Hickie, & Lagopoulos, 2014, for a review) and patients with Alzheimer’s disease (Crutcher et al., 2009). Although there has been a great deal of work looking at modeling patterns of fixations in adults, particularly looking at top-down and bottom-up influences (Itti et al., 1998, Xu et al., 2014), and there has been some important work examining how these influences develop during a child’s first months and years (e.g., Amso et al., 2014, Franchak et al., 2016, Frank et al., 2009), there has as yet been no systematic examination of different models applied to viewing dynamic scenes in school-age children compared with adults. We addressed this gap in the literature in this study.

### Development of fixation behavior

Certain viewing behaviors, such as looking at faces and stimuli with social relevance, develop so early in childhood that they appear to be largely innate. For example, it is well established that newborn babies preferentially track faces and face-like stimuli in simple displays (Farroni et al., 2005, Johnson et al., 1991). Using static (complex) images, several studies have shown that infants aged 6 months and older orient to faces in images that contain non-face distractors (Di Giorgio et al., 2012, Gliga et al., 2009, Gluckman and Johnson, 2013). Frank, Vul, and Saxe (2012) used videos of objects, faces, children playing with toys, and complex social scenes with young children aged 3–30 months. They showed further that facial and bodily features that have social relevance, such as eyes, mouths, and hands, capture infants’ and toddlers’ attention and that this capacity to direct their attention to the stimuli that are potentially the most socially informative increases with age. In another study, Frank, Amso, and Johnson (2014) reported an age-related increase in looking at faces in complex videos (clips from *Peanuts* [Charlie Brown] and *Sesame Street*) in 3- to 9-month-old infants, which correlated with increased attentional orienting using a visual search task, suggesting that the extent to which infants show social preferences may well be underpinned by their ability to detect socially relevant stimuli in otherwise complex dynamic scenes. Frank et al. (2014) finding is also consistent with a recent report showing that infants over 4 months of age tended to look first and longest at faces, whereas 4-month-olds tended to look at the most salient object in a display (Kwon, Setoodehnia, Baek, Luck, & Oakes, 2016).

Less is known, however, about the developments in fixation behavior that take place beyond early childhood. Kirkorian, Anderson, and Keen (2012) showed children (aged 1–4 years) and adults 20-min clips of television shows and found that younger children fixated more regions over a larger area than did older children. This variability was greatest immediately following scene cuts, which these authors proposed is due to an inability to suppress attention to irrelevant features (from the previous scene). More recently, Helo, Pannasch, Sirri, and Rämä (2014) examined differences in scanning behavior in adults and children aged 2–10 years and reported that fixation durations decreased and saccade amplitudes increased with age, at least with static images of naturalistic scenes, which these authors attributed to gains in general cognitive development. Similar age-related trends have been found in a preferential looking task, with eye movement response times falling with age from 1 to 12 years (Kooiker, van der Steen, & Pel, 2016). Interestingly, the response time to fixate highly salient targets reduced more rapidly with age than did fixations to less salient targets.

### Modeling natural fixation

One key question is precisely what is driving development in fixation behavior during childhood. Biologically inspired models have been developed to account for patterns of fixation within complex scenes and fall into two broad categories. *Saliency* models are driven by low-level (pixel-based) saliency and predict that eye movements are drawn to regions of visual information that differ locally in some basic feature (e.g., orientation, color) (Itti et al., 1998). In this scenario, fixation is mainly driven by a *bottom-up* process that relies primarily on sensory (rather than cognitive) processing (Xu et al., 2014). For simplicity, we refer to this class of model as “saliency” driven. The second class of models, *top-down* models, suggests that our eye movements are largely driven by cognitive or contextual factors. In this scenario, we expect that factors that affect top-down processing (e.g., age) will influence performance. Although age may also be considered a factor in bottom-up processing (as low-level sensory processing becomes more developed), this is unlikely to be the case for older children given that low-level sensory processes underlying acuity are adult-like by 3 years of age (Brown & Lindsey, 2009).

#### Saliency-based models

Most low-level models of fixation are based on Koch and Ullman’s (1985) and Itti et al. (1998) saliency model of visual attention in static images. These authors proposed that eye movements are preferentially driven by points of high image saliency, where the local statistics of an image patch differ from its surround. This model, and extensions of it, has been very influential (e.g., Baddeley and Tatler, 2006, Itti and Koch, 2000) and has spurred new analysis methods (e.g., Barthelmé, Trukenbrod, Engbert, & Wichmann, 2013), but it has only recently begun to incorporate the importance of features of dynamic images such as object motion and transformation. This is a major shortcoming because a reliance on static scenes is likely to minimize the relevance of ongoing semantic context such as social relevance and knowledge of cause and effect. We return to this point below.

#### Top-down models

Nonvisual factors influence interobserver variability in fixation patterns (particularly in response to dynamic stimuli). For example, when a video clip of people conversing is accompanied by a soundtrack that matches the visual content, observers are more accurate at localizing the face of the speaker (Coutrot & Guyader, 2014). Here, the sound needs to be understood as a human voice in order to drive eye movements toward the inferred speaker. But note that it is possible in some cases that the synchrony of sounds and visual transients (e.g., mouth movements) by themselves may drive eye movements, in which case audio can act as a more bottom-up influence. Other high-level processes have been shown to contribute to eye movements. For example, object “importance” (’t Hart, Schmidt, Roth, & Einhauser, 2013), social cues (faces or gaze; e.g., Birmingham, Bischof, & Kingstone, 2009), task instructions (Ballard and Hayhoe, 2009, Koehler et al., 2014), a person’s prior expectation about a scene (Eckstein, Drescher, & Shimozaki, 2006), and a person’s memory of a visual task (Ballard & Hayhoe, 2009) all can influence where a person allocates his or her attention in a visual scene and might not always be the region of highest saliency.

A recent analysis of eye movements by Xu et al. (2014) investigated the potential influence of both bottom-up and top-down factors during fixation. They categorized static images into different attribute qualities: “pixel attributes” (low-level features akin to saliency), “object attributes” (e.g., object size, eccentricity), and “semantic attributes” (e.g., whether an object is being looked at by an individual in the scene). They reported that semantic-level attributes that would reflect top-down processing—particularly objects being gazed at, faces, and text—influenced observer fixations more than lower-level saliency.

#### Dynamic stimuli

It is increasingly recognized that fixation during prolonged presentation of static visual scenes might not be representative of natural viewing behavior of complex dynamic scenes. Consequently, dynamic stimuli (e.g., clips, movies) are increasingly being employed to gauge visually guided behavior. Yet, this new approach brings with it a new set of complications. For example, Dorr, Martinez, Gegenfurtner, and Barth (2010) reported that the type of dynamic stimulus used can largely influence performance. They reported a higher degree of variability of eye movements between observers when watching natural movies as compared with commercial movies. This finding arises largely because commercial movies suffer more from “center of screen bias” (e.g., relevant objects are framed in the center of the shot) as well as an increase in temporal structure arising from frequent cuts, whereas Dorr and colleagues’ natural movies were approximately 20 s uncut and were generally shot from fixed camera positions, meaning that the ongoing action in the clip would not necessarily have occurred in the center of the screen. In another example, Mital, Smith, Hill, and Henderson (2010) reported that gaze clustering across observers viewing a dynamic scene is determined mainly by motion within the clip.

Although some of these studies have also quantified variability of eye movements, and studies have compared face and saliency models with adults and infants/toddlers (<4 years of age), there has been no systematic evaluation of different models that might account for changes in eye movement behavior in school-aged children viewing complex dynamic images. Furthermore, it has been shown that saliency is most influential in guiding the first fixations in static images, before top-down influences come into play (Parkhurst, Law, & Niebur, 2002). One expectation from this finding is that dynamic information may exacerbate the differences between early and later fixations. Specifically, we should expect that immediately after a scene cut, saliency will be more influential (we liken this to the first fixations in a static image), but that as the scene plays out, saliency influences will diminish. We also expect that, in general over the course of the clip, younger children’s viewing behavior will be better captured by a bottom-up saliency model than will that of adults.

### The current study

Here, we examined these changes in visual attention using a rich range of stimuli in which we measure the age-dependent variability in eye movements in school-age children relative to adults (taking into account scene cuts, which can lead to a change in semantic content in a film). We used longer-duration clips (3–6 min) of three different child-friendly movies (*Roadrunner* [cartoon], *Night at the Museum,* and *Elf*) to better examine the role of top-down (semantic) influences on eye movements as observers follow the story, and we sampled older children to allow these processes to have a greater influential role.

These methods enabled us to test two main accounts of visual attention across age groups: one that relies on low-level saliency models and one that relies on the presence of faces in the scene. Although much research has looked at eye movements in infants or young children (e.g., Franchak et al., 2016, Frank et al., 2009, Kirkorian et al., 2012), we focused here on older children to chart how the development of cognition and interest in social objects is reflected in eye movements. We hypothesized that (a) we would observe an increased consistency in fixations with age as reported in adults (e.g., Dorr et al., 2010), and consistent with reports in infants (e.g., Frank et al., 2009) and young children (e.g., Kirkorian et al., 2012), and that (b) a model predicting fixation behavior based on faces would outperform saliency models (Franchak et al., 2016, Frank et al., 2009).

## Method

### Participants

A total of 37 children from a range of ethnic backgrounds (identified by their parents as 19 Caucasian, 5 South Asian, 4 Black or Black/Caribbean, 3 Middle Eastern, 3 mixed race, and the remaining 3 unspecified) took part in the experiment during 1 week of “Brain Detectives,” a science club run at the UCL Institute of Education, University College London. Because several of the analysis methods we used to quantify performance (interobserver dispersion, cluster number, and normalized scanpath salience) depended on comparing fixation patterns *within groups* of individuals, it was not possible to treat age as a continuous variable in analyses. Therefore, we divided the children into two groups of approximately equal numbers and age ranges: “younger” (<10 years; *n* = 20, 9 girls, mean age = 7.8 years, range = 5.9–9.4) and “older” (≥10 years; *n* = 17, 12 girls, mean age = 11.9 years, range = 10.2–13.9). In addition, 10 adults (5 women, mean age = 31.9 years, range = 23.0–45.1) were tested at the UCL Institute of Ophthalmology, University College London. All participants reported normal or corrected-to-normal vision.

Written informed consent was obtained from the adults and from children’s parents prior to their or their children’s participation in the experiment. The experiment was approved by Institute of Education and Institute of Ophthalmology research ethics committees.

### Apparatus

Two identical systems were used for stimulus presentation and eye tracking. Calibration sequences and movies were presented using MATLAB (MathWorks, Natick, MA, USA) and the PsychToolbox (Brainard, 1997) running on Windows 7 PCs. Stimuli were displayed on LG W2363D LCD monitors (1920 × 1080 pixels, refresh rate = 60 Hz). The displays were calibrated using a photometer and linearized using lookup tables in software. Eye-tracking data were collected on two EyeLink 1000 systems with remote cameras (SR Research, Mississauga, Ontario, Canada) at 250 or 500 Hz. The reported average accuracy of the eye tracker is 0.5°, the spatial resolution is 0.05°, and the remote camera allowed for head movements of up to 22, 18, and 20 cm (horizontal, vertical, and depth, respectively). Viewing distance was approximately 57 cm, so that 1 pixel subtended approximately 1.6 arcmin and movies (at a resolution of 1708 × 960 pixels) covered 43.4 × 25.1° of visual angle. Children watched the videos individually while seated in a dimly lit room on a chair whose height could be adjusted so that their eyes were roughly level with the center of the screen. An experimenter was in the cubicle with them, seated at a different table, controlling the eye tracker. The experimenter explained the procedure to the children and informed them that they would be asked questions at the end of each clip. Adults viewed the videos at the Institute of Ophthalmology in a dimly lit room. In all cases, no chin rest was used.

### Stimuli

All participants watched two 5-min video clips and one 3-min video clip with their corresponding soundtracks to enhance comprehension of, and engagement with, movie content. One video was a cartoon (*Roadrunner*) and two others were taken from popular live-action children’s movies (*Night at the Museum* and *Elf*).

In terms of *shot segmentation*, a cut is an abrupt transition from one shot to another that greatly affects visual exploration (Garsoffky et al., 2007, Smith et al., 2012). In the following, analyses were performed on each individual shot (see Table 1). As in Coutrot, Guyader, Ionescu, and Caplier (2012), shots were automatically detected using a pixel-by-pixel correlation value between two adjacent video frames. We ensured that the shot cuts detected were visually correct.

**Table 1:** Stimuli characteristics.

	Video clip		
	*Elf*	*Night at the museum*	*Roadrunner*
Total duration (s)	333.2	366.5	204.8
Number of shots	105	93	38
Average shot duration (s) [*M* (*SD*)]	3.1 (2.2)	3.7 (3.2)	5.1 (3.6)

### Eye-tracking procedure

Calibration routines were run at the start and end of each of the three clips with a custom-made cartoon character (size = 1°) appearing at each of nine positions: the four corners, the four midpoints of the edges, and the center of the movie frame. Each data sequence was used to parse the *x*/*y* position signal into saccades and fixations/smooth pursuits with a custom algorithm based on Nyström and Holmqvist (2010) (see below). In adults, eye-tracking data were successfully recorded in 10 of 10 adults across all clips. For the children, no data could be collected for 2 of 37 children (both in the younger age group) because the eye tracker failed to locate and track their pupil. Intermittent loss of the eye-tracking signal (due to either body or head motion while watching the clips) led to data from a further 3 children (2 in the younger group and 1 in the older group) missing from two clips, and 6 children (1 from the younger group and 5 from the older group) had missing data from one clip.

### Data pre-processing

If the calibrations at the start and end of a clip were different (by visual inspection), data from the corresponding clip were not used. For Clip 1 (*Elf*), 6 younger and 6 older children were excluded; for Clip 2 (*Night at the Museum*), 11 younger and 7 older children were excluded; and for Clip 3 (*Roadrunner*), 10 younger and 9 older children were excluded. This left 10 adults, 14 younger children, and 11 older children with usable data for Clip 1; 10 adults, 9 younger children, and 10 older children for Clip 2; and 10 adults, 10 younger children, and 8 older children for Clip 3. Data where “start” and “end” calibrations matched but there was a loss of signal for part of the clip were used, with the corresponding signal-less sections cut out.

Saccades were identified based on the Nyström and Holmqvist (2010) iterative procedure that uses eye velocity and acceleration to set thresholds. Any period when velocity or acceleration exceeds an upper threshold for a minimum amount of time (10 ms) was classified as a saccade. The start- and end-points of the saccade were identified by going backward or forward in time until both the velocity and acceleration fell below a lower threshold. Saccades were identified to distinguish periods of fixation or smooth pursuit. The eye position data are missing during a blink, but there is also a period immediately before and after the blink when the dynamic occlusion of the pupil by the eyelid generates large, spurious motion signals in the eye-tracking data. The start- and end-points of blinks were identified using the lower threshold, as above, and all data during a blink were removed from the analysis.

Estimating eye-tracking data quality is critical because systematic differences in the quality of raw eye position data can create a false impression of differences in gaze behavior between groups. This is particularly true when comparing children and adults because children are more prone to postural change during testing than adults, leading to generally poorer or more variable eye-tracking data. For this reason, we assessed our eye-tracking data using the precision metric proposed by Wass, Forssman, and Leppänen (2014). This metric quantifies the degree to which eye positions are consistent between samples (the higher the metric, the less precise the eye data). For each participant, we averaged this metric across the three video clips for an overall measure of precision.

### Data analysis

Data were analyzed in two ways. First, we examined the variability between eye movements across observers for age-related changes, that is, an analysis based entirely on eye-tracking data (a *gaze-based analysis*). Second, we compared the eye-tracking data with the movie content using standard low-level (salience-based) models as well as a model based entirely on faces (a *content-based analysis*).

#### Variability between participants: Gaze-based analysis

As mentioned in the stimuli description, we performed our analyses on each frame of each individual shot.

##### Interobserver dispersion

To estimate the variability of eye positions between observers, we used a dispersion metric. This metric is commonly used in eye-tracking studies (Marat et al., 2009). For a given frame watched by *n* observers ((*x_i_, y_i_*) *i*∈[1, 2, ... n] the eye position coordinates), the dispersion *D* is defined as follows:

$$D(x\text{,}y)=\frac{1}{n(n-1)}\overset{n}{\underset{i=1}{\sum }}\overset{n}{\underset{\begin{array}{c} j=1\\ j\neq i \end{array}}{\sum }}\sqrt{{\left(x_{i}-x_{j}\right)}^{2}+{\left(y_{i}-y_{j}\right)}^{2}}\text{.}$$

The dispersion is the mean Euclidian distance between the eye positions of different observers for a given frame. The smaller the dispersion, the less scatter in the eye positions.

##### Cluster number

To quantify the number of points of interest attracting observers’ gaze, we performed a cluster analysis. For each frame, we clustered observers’ eye positions with the *mean shift* algorithm (Fukunaga & Hostetler, 1975). This algorithm considers feature space as an empirical probability density function. Mean shift associates each eye position with the closest peak of the dataset’s probability density function. For each eye position, mean shift defines a circular window of width *w* around it and computes the mean of the data points. Then, it shifts the center of the window to the mean and repeats the algorithm until it converges. This process is applied to every observer. All eye positions associated with the same density peak belong to the same cluster. The main advantage of mean shift compared with other popular clustering algorithms such as *k*-means is that it does not make assumptions about the number or shape of clusters. The only parameter to be tuned is the window width. Here, we took *w* = 100 pixels. Other *w* values between 50 and 200 pixels did not significantly change the results.

#### Comparison of models in dynamic visual information: Content-based analysis

##### Faces-based model

We compared variants of a saliency model against a faces-based model. For each clip, the faces were labeled using a custom MATLAB script. When a face first appeared on the screen, an ellipse was drawn around it. When the face moved during the scene (due to either person or camera movement), the position, orientation, aspect ratio, and size of the ellipse were updated in a small number of “keyframes” and interpolated in between times. This led to a relatively fast and accurate labeling of the whole clip. Points within these ellipses were set to 1, and outside they were set to 0, to produce a binary map that was then filtered with a three-dimensional spatiotemporal Gaussian filter (spatial *SD* = 1.06°, temporal *SD* = 26.25 ms) similar to that used by Dorr et al. (2010) to produce a “face map” akin to the saliency maps described below. Given the sparsity of visual information in the *Roadrunner* cartoon, for this clip only we also labeled objects in a similar manner, with the exception being that the outlines could be polygons as well as ellipses.

##### Saliency models

Three variants of the Itti et al. (1998) saliency model were applied to each movie: (a) Itti et al. (1998) and (b) Harel, Koch, and Perona (2006) graph-based visual saliency (GBVS) *with* motion/flicker channels and (c) Harel et al. (2006) GBVS *without* motion/flicker channels. All saliency models seek to extract regions that somehow differ from their surroundings. Itti et al. (1998) separated the image into three different channels based on luminance, color, and orientation, and within each channel they identified regions of the image that differ from their immediate surround. These individual maps were then linearly combined to produce an overall map highlighting unusual regions, with the implicit assumption that these regions are likely to draw our attention. The GBVS model similarly splits the image into separate channels (luminance, color, and orientation plus two dynamic channels, motion and flicker, based on differences between consecutive frames) and uses a graph-based approach to highlight unusual regions. Although the GBVS model is not as intuitive as the Itti et al. model, it has been shown to perform better for static images (Itti et al., 1998), and its incorporation of motion and flicker channels makes it appropriate for dealing with dynamic images.

##### Comparison of model performance

We used two methods of assessing the performance of each of the models. The first is the normalized scanpath salience (NSS), which has been extended to allow analysis of moving images (Dorr et al., 2010, Marat et al., 2009), and the second is the area under a receiver operating characteristic (ROC) curve (Green & Swets, 1966) as proposed for eye-tracking analysis by Tatler, Baddeley, and Gilchrist (2005).

##### Normalized scanpath salience

**Fig. 1:**
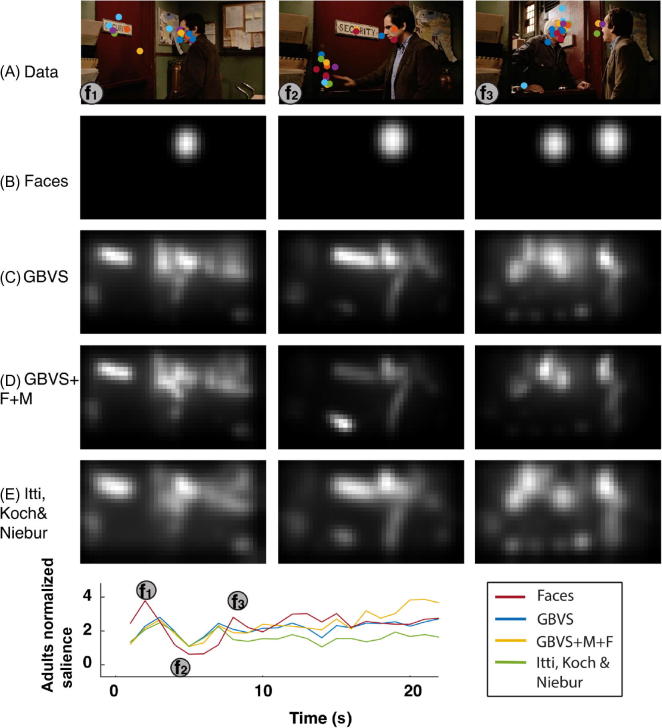
Sample frames and analysis from *Night at the Museum.* (A) Eye tracking from all participants (adults and children) shown as colored dots. (B–E) Outputs of faces-based model (B), graph-based visual saliency (GBVS) model (C), GBVS model with motion and flicker components (GBVS+F+M) (D), and Itti, Koch, & Niebur model (E). The bottom panel plots normalized salience for the adult group as a function of time. Note that in some cases (e.g., Frame f_2_), although a face is present, most eye movements followed the hand motion.

On a frame-by-frame basis, the salience (or face) maps were normalized by subtracting the mean and dividing by the standard deviation (face maps for frames in which no faces were present were set to 0; see Fig. 1). Regions where the model predicts a high probability of fixation will have positive values, and low-probability regions will be negative. The normalized saliency value for each fixation is then averaged across participants to give frame-by-frame model performance as well as across the duration of the movie to give an overall model performance value.

##### Correlation between outputs of a faces-based model and saliency models

To quantify any overlap between face maps and bottom-up saliency maps, we calculated the correlation between a faces-based model and saliency models. For each frame, we computed a pixelwise correlation between bottom-up saliency maps (Harel et al., 2006, GBVS and GBVS + motion/flicker; Itti et al., 1998) and face maps. Frames without a face have been discarded from the analysis.

##### Area under the ROC curve

The area under the curve (AUC) is often used as a measure of model performance. We threshold the saliency or face maps at different levels to find regions of predicted fixations for that particular threshold. By comparing these regions with where the actual fixations occurred, we can extract the proportion of “true positives” (proportion of fixations within the predicted region) and the proportion of “false positives” (pixels that the model highlighted but were not fixated). By varying the threshold between 0 and 1, we produce an ROC curve (see panel in Fig. 4 in Results). The area under this curve is used as a measure of model performance. A value close to 1 indicates that the model explains the data well, whereas chance performance is .50. We derived confidence intervals for the AUC values via bootstrapping. Although an AUC analysis has a well-defined upper bound of 1, it is more appropriate to extract an empirical upper bound from the eye-tracking data themselves. A low AUC score could be due to a poor model, high variability between the looking strategies of different people, or a combination of the two. A modification of Peters, Iyer, Itti, and Koch’s (2005) NSS can be used to overcome this problem (Dorr et al., 2010). The NSS is calculated by using a “leave one out” approach—taking the scanpaths of *n* − 1 observers, spatiotemporally blurring these, and summing and normalizing them to produce a map of where these *n* − 1 people fixated over time. This map is evaluated as a predictor of where the *n*th person fixates, using the area under an ROC curve as above. This process is repeated *n* times, once for each observer, and the NSS score is the average AUC value. We used a similar technique to compare both between and within groups, that is, using all of the younger children’s fixations to build a fixation map and using this map to predict either the older children’s or the adults’ fixations.

##### Statistical analyses

To compare our findings for different models, age groups, and movies, we performed one-way and two-way analyses of variance (ANOVAs) as appropriate. For any significant differences between conditions, we also performed pairwise comparisons using *t* tests with Bonferroni correction for multiple comparisons.

## Results

### Eye-tracking data variability

Using Wass et al. (2014) precision metric, we found no significant effect of age on precision: one-way ANOVA, *F*(2, 45) = 1.52, *p* = .23 (younger children: precision = .92, *SEM* = .19; older children: precision = .92, *SEM* = .19; adults: precision = .46, *SEM* = .03).

### Variability: Gaze-based analysis

#### Interobserver dispersion

**Fig. 2:**
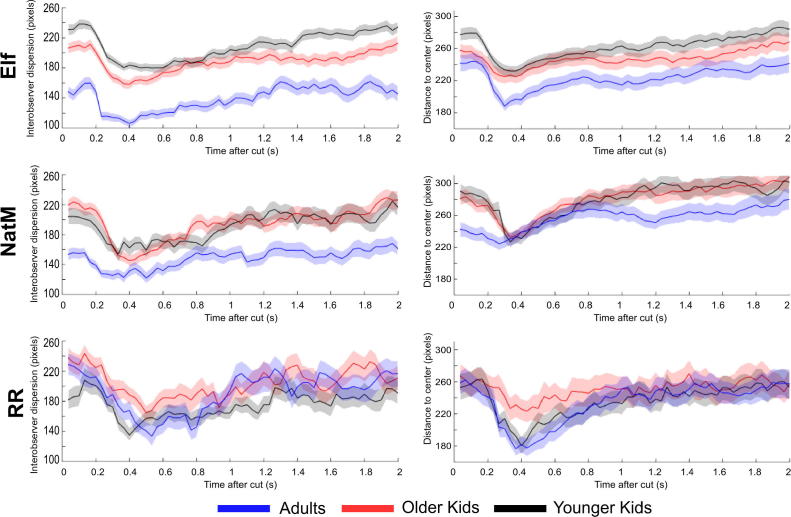
Temporal evolution of the interobserver dispersion across age group and clip for the first 2 s after a cut (left panels) and for the distance to the screen center (right panels). Values are averaged over the shots. Error bars correspond to ±1 standard error of the mean. NatM, *Night at the Museum*; RR, *Roadrunner*.

The shape of interobserver dispersion curves depicted in Fig. 2 (left panels) is conventional (Coutrot & Guyader, 2014). During the first 200 ms after a cut, dispersion is stable. During this period, observers’ gaze stays at the same locations as before the cut (latency period). Following this, dispersion decreases until 400 ms and slightly reincreases up to 1 s. This leads to the last stage, where the dispersion plateaus around a mean stationary value until the next cut. We ran a two-way ANOVA on interobserver dispersion with age (adults, older children, or younger children) and clip (*Elf*, *Night at the Museum,* or *Roadrunner*) as factors. There was a main effect of age, *F*(2, 707) = 20.76, *p* < .001, but not of clip, *F*(2, 707) = 2.87, *p* = .06. Post hoc Bonferroni tests showed that the dispersion was lower in adults than in older children, *t*(470) = −8.14, *p* < .001, or younger children, *t*(470) = −8.43, *p* < .001. There was no significant difference between the latter groups, *t*(470) = −0.29, *p* = .96. The interaction was also significant, *F*(4, 707) = 9.89, *p* < .001. For the *Roadrunner* clip, dispersion values were lower in younger children than in adults, *t*(74) = −2.20, *p* = .03, or in older children, *t*(74) = −2.68, *p* = .009. There was no difference between older children and adults, *t*(74) = 0.37, *p* = .71.

#### Distance to center

The global shape of distance to center is similar to the interobserver dispersion (Fig. 2, right panels). The stronger center bias around 200 ms after a cut has been reported previously (Wang, Freeman, Merriam, Hasson, & Heeger, 2012). It is due to a conjunction of factors, including the fact that the center of a scene is the optimal location to begin exploration (Tatler, 2007, Tseng et al., 2009).

We performed a two-way ANOVA on distance to center with age and movie as factors. There was a main effect of age, *F*(2, 707) = 5.33, *p* = .005, and movie, *F*(2, 707) = 10.69, *p* < .001. Post hoc Bonferroni tests showed that the distance to center was significantly lower in adults than in older children, *t*(470) = −3.39, *p* = .002, or in younger children, *t*(470) = −4.00, *p* < .001. There was no significant difference between the latter groups, *t*(470) = −0.61, *p* > .90.

#### Number of clusters

**Fig. 3:**
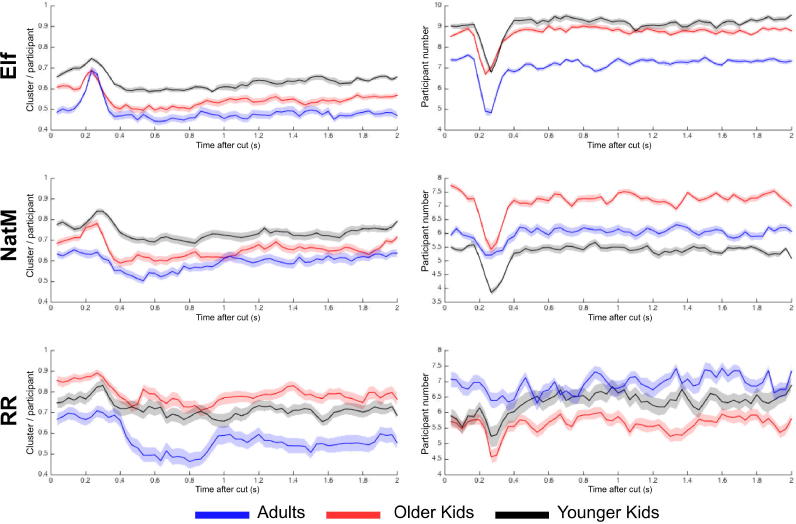
Temporal evolution of the number of clusters normalized by the number of observers, across age group and film, for the first 2 s following a cut (left panels) and for the number of observers (right panels). Values are averaged over all shots. Error bars correspond to ±1 standard error of the mean. NatM, *Night at the Museum*; RR, *Roadrunner*.

The number of clusters quantifies the number of points of interest attracting observers’ gaze. Because this number is likely to increase with the number of observers, we normalized it by the number of participants. The number of clusters is stable across time except for a brief increase at around 200 ms after the beginning of the shot (Fig. 3). This increase can be explained by looking at the right panel of Fig. 3, where the temporal evolution of the number of recorded observers is depicted. We clearly see a loss of approximately 20% of recorded participants between 200 and 300 ms. This might be due to blinks induced by sharp cuts, leading to a brief loss of eye-tracking signal. Because the number of clusters is normalized by the number of participants, a decrease in the latter logically causes an increase in the former.

**Fig. 4:**
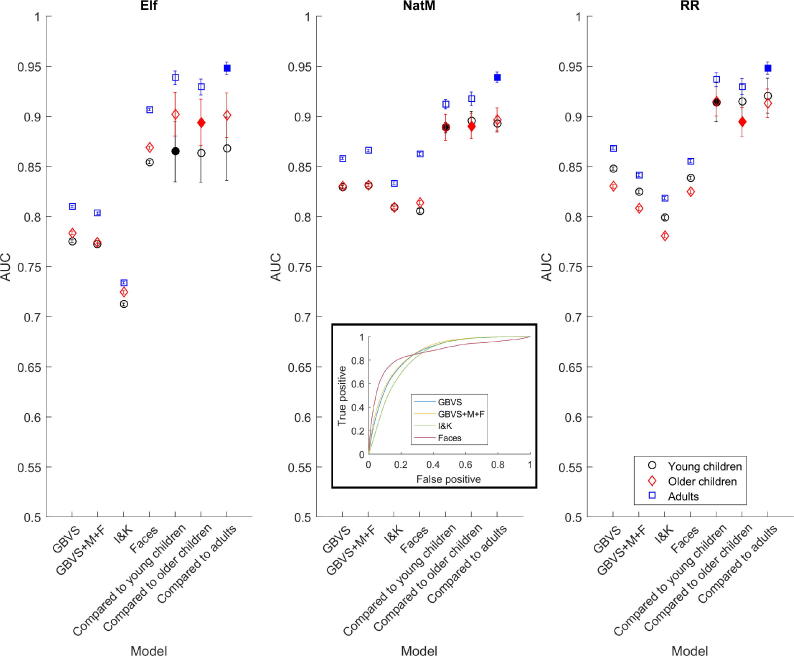
Area under the curve (AUC) measures as a function of clip and age group. GBVS, Harel, Koch, & Perona’s graph-based visual saliency model without motion and flicker components; GBVS+M+F, Harel, Koch, & Perona’s graph-based visual saliency model with motion and flicker components; I&K, Itti, Koch, & Niebur’s multichannel saliency model; Faces, our faces-based model. Solid symbols represent normalized scanpath salience conditions estimated against the same age group (e.g., solid black symbols represent young children compared with their own age group). Inset shows receiver operating characteristic curves for four models for *Night at the Museum* (NaTM). Error bars are ±1 standard error of the mean. RR, *Roadrunner*.

We ran a two-way ANOVA on the number of clusters normalized by the number of observers with age and clip as factors. There was a main effect of age, *F*(2, 707) = 90.87, *p* < .001, and clip, *F*(2, 707) = 91.59, *p* < .001. Post hoc Bonferroni tests showed that for *Elf* and *Night at the Museum,* the number of clusters was significantly lower in adults than in older children, *t*(394) = −5.26, *p* < .001, or in younger children, *t*(394) = −11.76, *p* < .001, and was significantly lower in older children compared with younger children, *t*(394) = −7.16, *p* < .001. For the *Roadrunner* clip, the number of clusters was still lower in adults than in older children, *t*(74) = −8.40, *p* < .001, or in younger children, *t*(74) = −4.83, *p* < .001, but surprisingly it was higher in older children compared with younger children, *t*(74) = 4.32, *p* < .001.

## Models

Fig. 4 shows the model performances and the within- and between-group scanpath similarities for the three clips, broken down by age group (younger children in black, older children in red, and adults in blue). We performed a three-way ANOVA on the AUC with age, clip, and model and their interactions as explanatory variables. Note that the models included in this analysis were the three salience models: Harel et al.’s (2006) GBVS model, either with (GBVS+M+F) or without (GBVS) motion and flicker components, and Itti et al.’s (1998) multichannel saliency model (I&K). The faces-based model and the within-group NSS model showed that there were significant differences in AUCs across age group, *F*(2, 44) = 158.77, *p* < .001, clip, *F*(2, 44) = 124.92, *p* < .001, and model, *F*(4, 44) = 520.43, *p* < .001, as well as their interactions [Age × Movie, *F*(8, 44) = 8.37, *p* < .001; Age × Model, *F*(8, 44) = 4.06, *p* = .008; Movie × Model, *F*(8, 44) = 67.22, *p* < .001]. Post hoc Bonferroni tests showed that there were significant differences between the adults’ data and both the younger and older children’s data [younger *t*(16) = 15.30 and older *t*(16) = 15.60, both *p*s < .001], but there was no significant difference between the two children’s groups, *t*(16) = 0.26, *p* > .90). Post hoc Bonferroni tests also showed that there were significant differences between the results for *Elf* and each of the other two clips [*Elf* vs. *Night at the Museum, t*(16) = 13.70, and *Elf* vs. *Roadrunner, t*(16) = 13.60, both *p*s < .001], but not between *Night at the Museum* and *Roadrunner, t*(16) = 0.12, *p* > .90. Similarly, there were significant differences between each of the models except for the two GBVS models (with and without motion and flicker) [GBVS vs. GBVS+M+F: *t*(16) = 3.00, *p* = .085; all other pairwise model comparisons: *t*(16) > 7 and *p* < .001].

It is clear that for all three clips, the models predict the adults’ data better than the children’s data. A three-way ANOVA comparing the within- and between-age group NSS data for the three clips showed that there were significant differences in age groups in terms of how well their data *could be predicted* by data from another group (including its own), *F*(2, 26) = 27.80, *p* < .001, with post hoc Bonferroni tests showing that this was due to adults’ data being better predicted than those of either group of children [adults vs. younger children: *t*(20) = 7.00, *p* < .001; adults vs. older children: *t*(20) = 5.70, *p* < .001; younger children vs. older children: *t*(20) = 1.40, *p* = .5610]. However, there were no significant differences in how well each age group *could be used to predict* another age group (including its own), *F*(2, 26) = 1.73, *p* = .203.

### Correlation coefficients between the faces-based and salience models

We found that correlations between the faces-based model and the bottom-up salience models were lowest for *Elf* (*M* = .31, *SD* = .001), followed by *Night at the Museum* (*M* = .35, *SD* = .001) and *Roadrunner* (*M* = .50, *SD* = .002). By way of comparison, the mean correlation coefficients between the maps of two bottom-up saliency models (Harel et al., 2006 [GBVS]; Itti et al., 1998 [I&K]) were .88, .90, and .87 for the respective three movies.

**Fig. 5:**
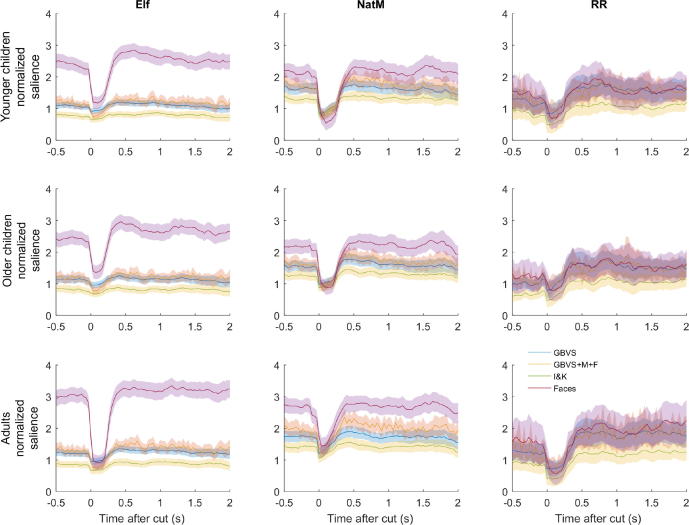
Normalized salience as a function of clip and age group for three versions of low-level saliency and the faces model. Data are aligned to 500 ms proceeding cuts in the clips. Error bars are 95% confidence intervals. NatM, *Night at the Museum*; RR, *Roadrunner*.

Fig. 5 shows the normalized saliencies in the 2 s after a cut (averaged across all cuts in each clip and all observers in each age group; shaded regions show the 95% confidence intervals). All models perform relatively poorly immediately after a cut and rise to a peak at about 500 ms. The difference between the faces-based model and the salience models is most pronounced for *Elf*.

## Discussion

We tracked the eye movements of children and adults viewing dynamic stimuli (movie and cartoon clips). We performed two types of analyses on the resulting data: comparing fixation within and between age groups (gaze-based analysis) and examining how well fixation could be predicted from low-level (salience) or top-down (faces) features of the movies (content-based analysis). We report the following results. First, variance between eye movements decreases with age, consistent with comparison of eye movements in infants and young children (Franchak et al., 2016, Kirkorian et al., 2012). Second, all models that we tested predicted adults’ performance better than children’s performance, again suggesting greater homogeneity in eye movements in adults. Third, a face-based model performs at least as well as the low-level saliency models and significantly outperforms them for one of our clips (*Elf*). This difference in performance among the three clips highlights the importance of using different types of stimuli in testing for visual attention and is consistent with Dorr et al. (2010), who reported that interparticipant variability in adult eye movements is greater using natural stimuli than using commercial clips.

For gaze-based analyses, the results showed that adults’ data were less variable than children’s data. The interobserver dispersion and number of clusters per person were lower for adults than for younger and older children, suggesting that adults look at a smaller number of objects or areas of interest when fixating a dynamic scene. The NSS analysis shows that the adults’ data are better predicted by other people’s data [whether those people are adults (within age groups) or children (between age groups)] than the children’s data. However, the adults’ data do not predict the children’s data (between) any better than the children’s data predict themselves (within). These findings suggest that although children on average look at more areas of interest in a scene (i.e., larger dispersion and numbers of clusters), there is significant overlap in the regions children and adults find most interesting. Because young children (4 or 5 years) have been found to have difficulty in maintaining accurate fixation (Kowler & Martins, 1982), the fixation heat maps for different age groups would be expected to be flatter for children (although they may have peaks in the same areas if they were looking at the same things as adults). This may account for some of the variability seen with our younger age group (the youngest child was 6 years old, only slightly older than the 4- and 5-year-olds in Kowler and Martins’s [1982] study), but given that fixation accuracy is likely to be more adult-like for the older children (10–14 years), this would not explain most of our results.

Two recent studies have extended eye-tracking analysis to dynamic stimuli in children. Kirkorian et al. (2012) showed 1-year-olds, 4-year-olds, and adults a 19.5-min clip of *Sesame Street* and performed a gaze-based analysis. They reported reduced variability with age that they linked to increased influence of top-down mechanisms. Our results are largely consistent with this finding when extended to children in their teens (older child group). Interestingly, we also found that roughly 200 ms after a shot, there is an increase in the number of clusters (in all age groups) associated with a corresponding decrease in the number of participants. The most likely explanation for this is that scene cuts lead to eye blinks (Nakano, Yamamoto, Kitajo, Takahashi, & Kitazawa, 2009). Franchak et al. (2016) showed adults and young infants (up to 24 months) a 60-s clip of *Sesame Street* and also looked at interparticipant variability in eye movements. They reported that younger infants’ eye movements were weakly correlated with those of adults but that this interparticipant correlation increased with age (24 months). Furthermore, correlations between adults and infant groups were no greater than correlations within the infant group, which is consistent with the data presented here.

For content-based analyses, we found that extending static models of faces (Frank et al., 2012) to dynamic stimuli leads to performance as good as, or better than, any of the low-level salience models of eye movements that we implemented (Harel et al., 2006, Itti et al., 1998). Comparing performance of the different models, we found that the Itti et al. (1998) model generally performed poorest and the faces-based model performed much better than the saliency models for *Elf* and is approximately as good as the best-performing salience model for *Night at the Museum* and *Roadrunner.* We also found that when there was only a weak correlation between our face maps and the low-level salience maps (as in *Elf*), there was a significant performance difference between the face and saliency models, with faces being more predictive of gaze behavior. If the faces based model performed well simply as a result of faces being highly salient parts of the image, we would expect the faces-based model to perform best when the correlation between faces and salience is highest. This is not what we observed. Therefore, we do not believe that the impressive performance of the faces-based model is simply due to faces being more salient than other objects. Interestingly, the GBVS model, which explicitly builds in motion and flicker components (and so might be expected to perform better on dynamic movies), does not appear to outperform the GBVS model that omits them. Indeed, for the cartoon clip, the opposite pattern of results was observed. This may be due to the style of animation, whereby a sparse scene made up of large blocks of uniform color typically contains one or two foreground objects of interest that are static in the frame (but moving in the world) and a number of (irrelevant and physically static) background objects that move across the screen behind them. It is worth noting that, surprisingly, the low-level models perform relatively well on these complex dynamic stimuli (average AUC = 80% vs. average AUC for faces-based model = 85%). A possible explanation for the lack of superiority of the face-based model for two of the three clips is that movies often contain more than one person in a scene, and this increases the number of possible face targets (also suggested by Franchak et al., 2016).

Surprisingly, we found no clear spike in performance for the salience models shortly after a cut, which may have been expected in light of findings for static images where salience performs particularly well for the first fixation after an image is shown (Carmi and Itti, 2006, Parkhurst et al., 2002), although other authors argue that the effect is mainly an artifact of a center bias. In our results, there is no clear evidence of a decline in saliency model performance over time, which often occurs for dynamic stimuli (Carmi and Itti, 2006, Marat et al., 2009). It is likely that in our dynamic stimuli, the constant appearance of new salient regions promotes bottom-up influences at the expense of top-down strategies, inducing a stable consistency between participants over time. Overall, our results are consistent with earlier reports using young infants. For example, Kwon et al. (2016) reported an increase in the eye movements to faces in static images, and Frank et al. (2012) found an increase in fixations to socially relevant information in brief videos. Recently, Franchak et al. (2016) compared consistency of eye movements with saliency and the presence of faces in 1-min clips from *Sesame Street,* and although they reported that on average fixations were to the top quartile of salient regions, they noted that a model that relies on both saliency and faces accounts for (only) 41% of the variance in infants’ eye movements. However, the different age ranges, stimuli, and analyses in our study and theirs mean that we cannot directly compare our results.

It is noteworthy that our procedure relies exclusively on modeling/quantifying eye-tracking behavior and, as such, is agnostic as to what may underlie these age-related changes. We postulate that most changes will reflect attentional orienting rather than motor or visual immaturities because we are not testing young infants (Farber & Beteleva, 2005). In fact, the youngest children tested were 6 years old. Although saccade latencies decrease with age (Fukushima et al., 2000, Salman et al., 2006), their accuracy and peak velocity is adult-like by 8 years of age. Smooth pursuit is age dependent. However, for targets moving at roughly 15°/s or lower, children’s pursuit of targets is adult-like (age 5 years and over) (Ego, Orban de Xivry, Nassogne, Yüksel, & Lefèvre, 2013). Given that, other than brief sections of the *Roadrunner* cartoon, most clips did not contain rapidly moving objects, we believe that any smooth pursuit immaturities would have a minimal influence on our results.

Finally, we note that the dependence of our outcomes on the particular movie sample highlights the importance of using stimuli that vary in their semantic/saliency content. This will be particularly important when applying such techniques to study pseudo-naturalistic fixation behavior in clinical populations. For example, the presentation of people with autism spectrum disorder differs widely, and it may be that patterns of abnormal fixation in some classes of movies can provide pointers to classification of autism spectrum disorder subtypes.

In summary, we have examined age-related changes in children and adults’ eye movements to dynamic visual scenes, namely popular child-appropriate cartoons and movies. We extended previous work in infancy and early childhood and showed that (a) variance in eye movements decreased with age; (b) a faces-based model outperformed saliency models, even for the youngest children; and (c) when there was an increased differentiation between faces and salient regions in a scene, participants looked more to the faces as in *Elf.* These findings shed light on the nature of visual attention during development and act as an important reference for understanding how such attention may develop differently in individuals with neurodevelopmental conditions.
